# Development and validation of a risk prediction model for cage subsidence after instrumented posterior lumbar fusion based on machine learning: a retrospective observational cohort study

**DOI:** 10.3389/fmed.2023.1196384

**Published:** 2023-07-21

**Authors:** Tuotuo Xiong, Ben Wang, Wanyuan Qin, Ling Yang, Yunsheng Ou

**Affiliations:** ^1^Department of Orthopedics, The First Affiliated Hospital of Chongqing Medical University, Chongqing, China; ^2^Plastic Surgery Hospital, Chinese Academy of Medical Sciences and Peking Union Medical College, Beijing, China; ^3^Department of Breast and Thyroid Surgery, The First Affiliated Hospital of Chongqing Medical University, Chongqing, China

**Keywords:** spine surgery, fusion, risk factors, prediction model, degenerative disease, cage subsidence

## Abstract

**Background:**

Interbody cage subsidence is a common complication after instrumented posterior lumbar fusion surgery, several previous studies have shown that cage subsidence is related to multiple factors. But the current research has not combined these factors to predict the subsidence, there is a lack of an individualized and comprehensive evaluation of the risk of cage subsidence following the surgery. So we attempt to identify potential risk factors and develop a risk prediction model that can predict the possibility of subsidence by providing a Cage Subsidence Score (CSS) after surgery, and evaluate whether machine learning-related techniques can effectively predict the subsidence.

**Methods:**

This study reviewed 59 patients who underwent posterior lumbar fusion in our hospital from 2014 to 2019. They were divided into a subsidence group and a non-subsidence group according to whether the interbody fusion cage subsidence occurred during follow-up. Data were collected on the patient, including age, sex, cage segment, number of fusion segments, preoperative space height, postoperative space height, preoperative L4 lordosis Angle, postoperative L4 lordosis Angle, preoperative L5 lordosis Angle, postoperative PT, postoperative SS, postoperative PI. The conventional statistical analysis method was used to find potential risk factors that can lead to subsidence, then the results were incorporated into stepwise regression and machine learning algorithms, respectively, to build a model that could predict the subsidence. Finally the diagnostic efficiency of prediction is verified.

**Results:**

Univariate analysis showed significant differences in pre−/postoperative intervertebral disc height, postoperative L4 segment lordosis, postoperative PT, and postoperative SS between the subsidence group and the non-subsidence group (*p* < 0.05). The CSS was trained by stepwise regression: 2 points for postoperative disc height > 14.68 mm, 3 points for postoperative L4 segment lordosis angle >16.91°, and 4 points for postoperative PT > 22.69°. If the total score is larger than 0.5, it is the high-risk subsidence group, while less than 0.5 is low-risk. The score obtains the area under the curve (AUC) of 0.857 and 0.806 in the development and validation set, respectively. The AUC of the GBM model based on the machine learning algorithm to predict the risk in the training set is 0.971 and the validation set is 0.889. The AUC of the avNNet model reached 0.931 in the training set and 0.868 in the validation set, respectively.

**Conclusion:**

The machine learning algorithm has advantages in some indicators, and we have preliminarily established a CSS that can predict the risk of postoperative subsidence after lumbar fusion and confirmed the important application prospect of machine learning in solving practical clinical problems.

## 1. Introduction

Posterior lumbar interbody fusion is the treatment of lumbar disc herniation, lumbar spinal stenosis, and lumbar spondylolisthesis, which is the most commonly used surgical procedure (1). Inserting a cage to maintain the intervertebral space height is an important step in lumbar fusion. But long-term follow-up after surgery shows that the intervertebral height may be lost. This can lead to instrumentation failure, pseudoarthrosis, kyphotic deformity, adjacent-segment disease, and loss of foraminal height, any of which can lead to recurrent nerve root impingement and radicular pain (2–4). And studies have shown that cage subsidence is associated with postoperative revision surgery, and the rate of revision is significantly higher in patients with high-grade cage subsidence after fusion, there are some studies showed the rate of cage subsidence can reach 38% (5–7). Previous clinical studies have shown risk factors of cage subsidence (2–5, 8). But currently, it is rare to comprehensively use multiple relevant risk factors to predict and analyze the risk of subsidence. Also, it is difficult to predict it accurately affected by complex risk factors (9–13). The most known used method for prediction in clinical practice is the scoring system based on logistic regression analysis (14–16). But as a classic statistic model, logistic regression-based methods may be insufficient in making full use of risk factor information (17–20). The emergence of ML (machine learning) has brought a turning point for improving this problem. It can detect the details of data features that human cannot, also reflected in its ability to efficiently handle complex nonlinear data (21, 22), and enable more efficient diagnosis (23).

Machine learning is one kind of computer algorithm, but its high-speed development recently makes it considered to bring about the fourth industrial revolution (24). Such as the widely used face recognition technology, which based on the convolutional neural network (CNN) algorithm (25, 26). The reality of self-driving cars is also highly dependent on deep learning algorithms (27). The most exciting thing is that Alpha Go based on the DL algorithm successfully beat master in Go chess (28, 29). And this is unimaginable without machine learning algorithms, because of the huge search space of Go, far beyond the capabilities of traditional methods such as the exhaustive method (30, 31). Different from the Deep Blue computer, which is also famous for beating humans, the underlying technology involved in Alpha Go, namely machine learning technology, is extremely versatile and can be extended to many application fields (32). Following the breakthrough of the DL in the field of Go, the alphafold also based on DL, significantly improved the accuracy of protein structure predictions that have been slowly thriving for decades (33, 34). So, machine learning has revolutionary value in the development of ‘Future Medicine’ (35).

Recently several papers focusing on the application prospects of ML technology in the future medical field had published (36, 37). Machine learning-related technologies have achieved remarkable results in several medical fields. In terms of risk prediction of complications, studies by Tomasev (38, 39) show that ML can effectively predict the risk of chronic kidney disease with diabetes, which facilitates early intervention for patients. It has also shown significantly better diagnostic performance than humans in image diagnosis: Hannun et al. (40) applied deep networks to ECG data and showed that the machine learning-based model can effectively tell ECG. But in general, the current attempts to combine medical practice with machine learning technology are still insufficient.

Therefore, the purpose of this study is to synthesize multiple risk factors to the traditional stepwise regression model and the machine learning model, respectively, to establish a scoring system, so as to predict the probability of cage subsidence after surgery, and further evaluate and compare whether they can effectively predict the risk after instrumented posterior lumbar fusion surgery.

## 2. Materials and methods

### 2.1. Case data

This study retrospectively collected the medical records and imaging data of 59 patients (73 surgical segments) who were admitted to the hospital from October 10, 2014, to December 29, 2019. Inclusion criteria: (1) Performed posterior lumbar fusion and implanted a cage. (2) The follow-up lasted for more than half a year. (3) Clinical diagnosis of lumbar disc herniation or lumbar spondylolisthesis. Exclusion criteria: (1) Patients with tumors, infections, and spinal fractures. (2) Patients with incomplete imaging data.

### 2.2. Imaging measurement methods

**Figure 1:**
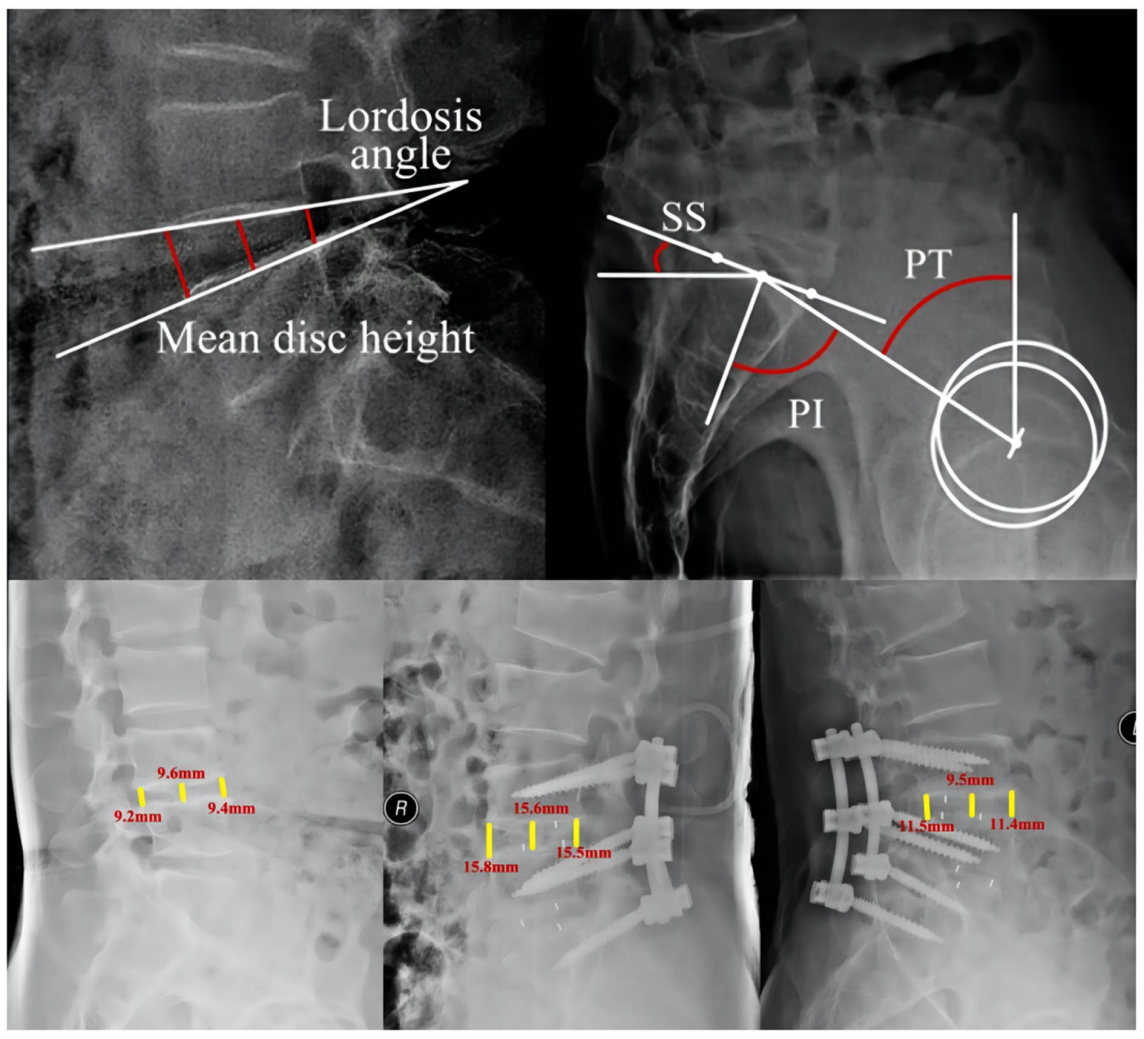
Diagram of the measurement of imaging data. PI, Pelvic incidence; SS, sacral slope; PT, pelvic tilt.

To measure the distance and angle of follow-up imaging data, respectively, by the Carestream imaging software (Figure 1). The height of the disc is defined as the mean of the front and middle and posterior intervertebral space. The height at the last follow-up less than 2 mm from 7 days after surgery was defined as cage subsidence (41, 42). The spinal and pelvic parameters were also collected: (1) Lumbar Segmental Lordosis: the cobb between the lower and upper end plate of the surgical segment, (2) Pelvic Incidence (PI): the angle between the line drawn from the femoral head axis to the midpoint of the sacral plate and the line perpendicular to the sacral plate, (3) Pelvic Tilt (PT): the angle between the vertical and the line drawn from the femoral head axis to the midpoint of the sacral plate, and (4) Sacral Slope: the angle between the sacral plate and the horizontal (43).

### 2.3. Principles of GBM model construction

Gradient boosting machine (GBM), which belongs to boosting algorithm, is a machine learning algorithm that can be used to deal with regression and classification problems (44). Promote. The simple principle of the algorithm can be understood as the establishment of many classification models by combining a series of weak, low-cost, and complex classifiers (weak learner), which are continuously adjusted through each classification, and finally the model is optimized (45). Model Averaged Neural Network (avNNet), the neural network is a machine learning algorithm that contains three layers (input layer, hidden layer, output layer), the hidden layer contains hidden neuron nodes, these neurons accept the input layer and output layer information at the same time. Initially, the neuron functions of the neural network was randomly distributed, but by continuously learning the sample features, the neurons constantly modify their parameters to achieve an accurate classification performance (46).

### 2.4. Surgical methods

The patient is placed in a prone position under general anesthesia, the surgical segment is positioned by C-arm before the operation, the anatomical structures at all levels are exposed in turn, and the pedicle screws are drilled and implanted. After the C-arm confirms the stability of the screws, choose an appropriate length of titanium rod, bend it into a physiological curvature, and lock the nut. Decompress and release the nerve root, the intervertebral disc tissue was removed, scrape the cartilage terminal plate to the vertebral end plate with a curette, and the intervertebral space was flushed. The allogeneic bone is taken and wrapped in a cage of suitable size and inserted into the intervertebral space. Drainage tubes should be placed, the close the wound after confirming that the cage is in a good position under fluoroscopy.

### 2.5. Modeling methods

Firstly, SPSS 21.0 software was used to conduct univariate analysis on the suspected cage subsidence-related factors selected in this study. The variables with *p* < 0.05 in the univariate analysis were subjected to stepwise regression, and the variables were screened based on the AIC principle (47). The selected variables were incorporated into the stepwise logistic regression and machine learning models. The code implementation involved in this study was done under R version 4.1.0.^1^ First, use the install. Packages (“*”) command to complete the installation of the integrated machine learning package caret (48), the ROC curve drawing package pROC (49). And use the createDataPartition command and follow 0. 75: 0.25 scale the original data set into a training set and a validation set. Then use the training command in the caret package (48) to train the model for statistically different variables (as described in the Results section) in the training set, use the glm function to build a logistic regression model, and finally use the trained model to train the training set. And the validation set is predicted (predict command), and the confusion matrix command is used to obtain the confusion matrix of the predicted results, as well as the specificity, sensitivity, F1 value and other indicators. The receiver operating curve (ROC) is drawn by the pROC package (49), and the core modeling code is shown in Supplementary material.

### 2.6. Statistical methods

It is statistically significant to use the t-test to test the difference between the sedimentation group and the non-sedimentation group, and if the variance was uneven, the Mann–Whitney *U* test was used. The results were expressed as mean ± standard deviation, and the counting data were expressed as frequency. The Chi-square test or Fisher’s precision test was used to test whether the frequency difference between groups was statistically significant, and *p* < 0.05 was used as the threshold for statistical significance.

The work has been reported in line with the STARD criteria (50).

## 3. Results

### 3.1. Basic information

**Figure 2:**
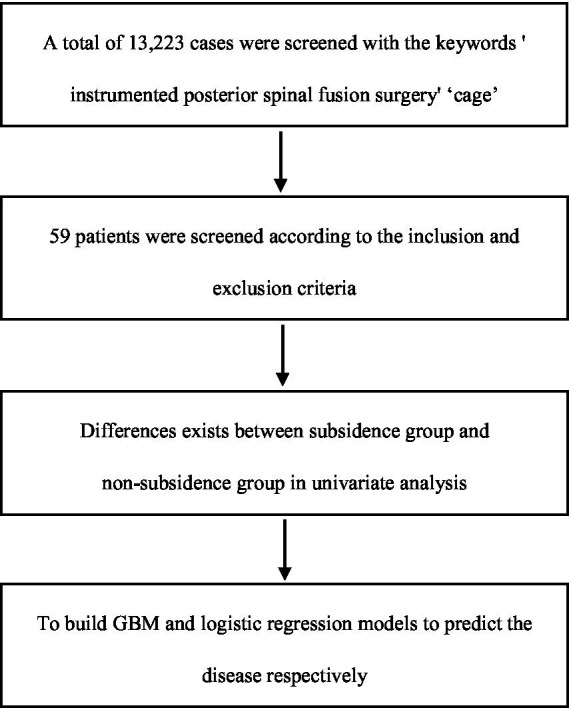
Research design.

The research process is shown in Figure 2. The basic information about the patients is shown in Table 1. There are 30 surgical segments from men and 43 from women, of which 39 are in the subsidence group, and 34 are in the non-subsidence group. The incidence rate was 53.42%, and there was no significant statistical difference between the subsidence group and the non-subsidence group in the basic characteristics (age, gender) of the patients (age: *p* = 0.982, gender: *p* = 0.643).

**Table 1:** Single factor analysis results ($\bar{x}\pm s$).

Characteristics	Subsidence group (*n* = 39)	Non-subsidence group (*n* = 34)	t/$x^{2}$-value	Value of *p*
Age	52.57±16.24	53.82 ±13.39	0.023	0.982
Gender				
Male	17	13		
Female	22	21	0.215	0.643
Segment of cage insertion (*n*)				
L4-L5	16	20		
L5-S1	23	14	2.302	0.129
Fusion segments (*n*)				
One	24	15		
Two				
Preoperative intervertebral space height (mm)	15			
11.68 ± 2.71	19	2.215	0.137	
		9.28 ± 3.42	−3.343	**0.001**
Postoperative intervertebral space height (mm)	15.92 ± 2.92	13.22 ± 3.07	−3.848	**<0.001**
Preoperative L4 lordosis (°)	10.88 ± 5.28	9.19 ± 5.23	−1.37	0.175
Preoperative L5 lordosis (°)	18.54 ± 6.44	17.68 ± 7.94	−0.513	0.61
Postoperative L4 lordosis (°)	12.97 ± 5.4	10.47 ± 5.03	−2.042	**0.045**
Postoperative L5 lordosis (°)	19.8 ± 5.12	18.64 ± 6.25	−0.865	0.39
Postoperative PT (°)	21.74 ± 8.28	26.36 ± 8.82	2.309	**0.024**
Postoperative SS (°)	38.16 ± 8.53	33.99 ± 8.47	−2.088	**0.04**
Postoperative PI (°)	59.89 ± 11.69	60.36 ± 12.29	0.165	0.87

The bold values are provided in statistical significance.

In terms of surgical segments, a total of 37 L5-S1 and 36 L4-L5 fusion segments were collected, of which the fusion subsidence rate of the L5-S1 segment was 62.16%, and the subsidence rate of the L4-L5 segment was 44.44%. There was no significant difference in the sedimentation rate between the two segments (*p* = 0.129). Among the collected cases, 39 cases were single-segment fusion, and 34 were double-segment fusion. The difference was not statistically significant (61.54% vs. 44.12%, *p* = 0.137).

As shown in Table 1, compared with the non-subsidence group, the subsidence group had statistically significant differences in preoperative/postoperative intervertebral space height, postoperative L4 segment lordosis angle, and postoperative PT and SS variables. Compared with the non-subsidence group, the subsidence group had higher preoperative/postoperative intervertebral space height (11.68 ± 2.71 vs. 9.28 ± 3.42 preoperatively, 15.92 ± 2.92 vs. 13.22 ± 3.07 postoperatively, *p* < 0.001), indicating that patients with higher preoperative intervertebral space height, excessive intraoperative distraction, and inappropriate cage size may be risk factors for postoperative cage subsidence.

In terms of postoperative lumbar spine angle, the postoperative L4 segment lordosis of the subsidence group was significantly higher than that of the non-subsidence group (12.97 ± 5.4 vs. 10.47 ± 5.03, *p* = 0.045), and this indicator did not achieve a statistically significant difference in preoperative measurements (10.88 ± 5.2 8 vs. 9.19 ± 5.23, *p* = 0.175), suggesting that the effect of surgery on the patient’s lumbar curvature (such as the angle of bar bending) may be a risk factor for postoperative sedimentation.

In terms of pelvic parameters, patients in the subsidence group have a smaller Pelvic Tilt (PT) compared to the non-subsidence group (21.74 ± 8.28 and 26.36 ± 8.82, *p* = 0.024) and larger Sacral Slope (SS) (38.16 ± 8.53 and 33.99 ± 8.47, *p* = 0.04), but there was no statistical difference in Pelvic Incidence angle (Pelvic Incidence, PI) (59.89 ± 11.69 and 60.36 ± 12.29, *p* = 0.87).

### 3.2. Sedimentation risk score based on stepwise logistic regression

**Figure 3:**
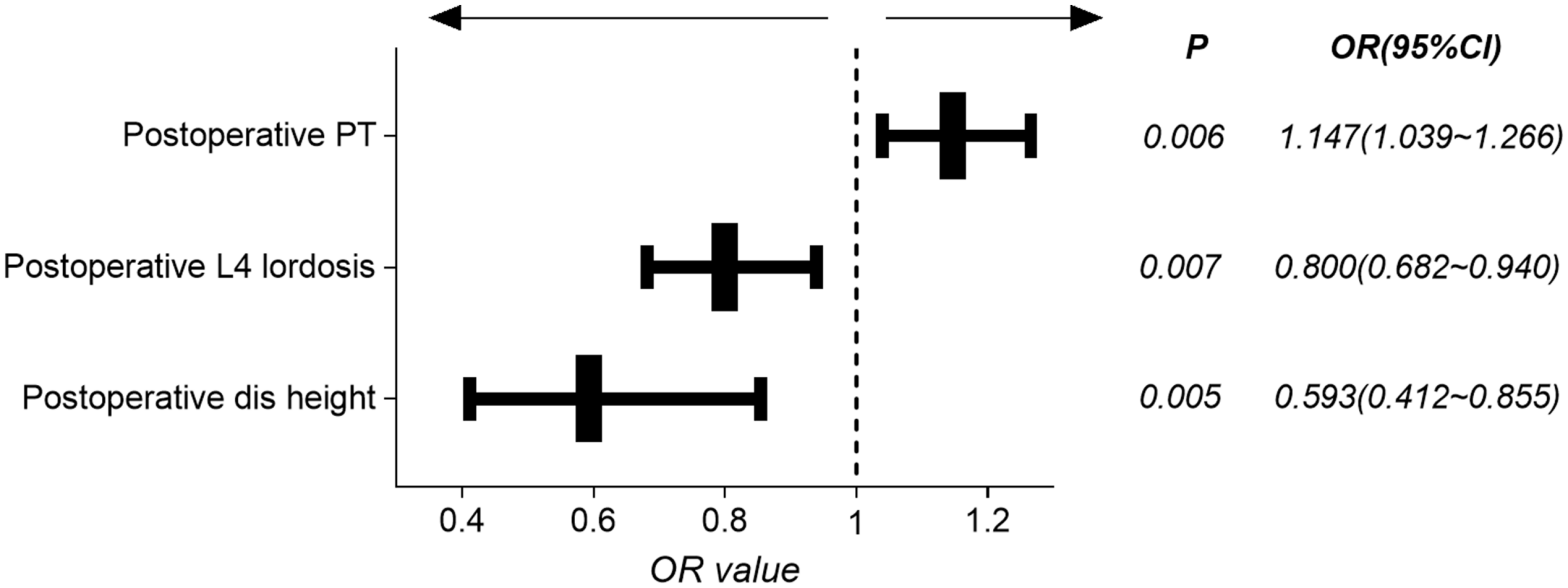
The forest map of influencing factors.

Combining clinical experience and univariate analysis results, we included preoperative/postoperative intervertebral space height, postoperative L4 lordosis angle, postoperative PT and postoperative SS variables, using the step (model, direction = ‘both’) command, based on The Akaike information criterion (AIC) (47) and the forward-backward stepwise regression method (51) are used to initially screen the above variables. The AIC criterion was founded by Akaike Hiroji, a variable screening method based on the concept of information entropy to balance model complexity and goodness of fit (47). The results are shown in Table 2, since the *p-*value of the preoperative intervertebral space height and the postoperative SS factor is greater than 0.05 under AIC optimization, we ruled out these factors and carried out secondary modeling. As shown in Table 3, postoperative PT is an independent protective factor for cage subsidence, while postoperative L4 lordosis angle and postoperative intervertebral space height are independent risk factors for cage subsidence. Patients with lower postoperative PT, higher postoperative L4 lordosis angle, and higher postoperative intervertebral space height had a higher risk of cage subsidence, and the forest map of influencing factors of cage subsidence is shown in Figure 3.

**Table 2:** First stepwise regression results.

Factors	Regression coefficients	OR	95%CI	*z*-value	Value of *p*
Preoperative dis height	−0.052	0.95	0.699–1.291	−0.33	0.741
Postoperative dis height	−0.494	0.61	0.394–0.944	−2.218	**0.027**
Postoperative L4 lordosis	−0.21	0.811	0.69–0.951	−2.568	**0.01**
Postoperative PT	0.131	1.139	1.032–1.258	2.58	**0.01**
Postoperative SS	−0.074	0.929	0.848–1.017	−1.589	0.112

The bold values are provided in statistical significance.

**Table 3:** Regression results after excluding nonsignificant factors.

Factors	Regression coefficients	OR	95%CI	*z*-Value	Value of *p*
Postoperative dis height	−0.522	0.593	0.412–0.855	−2.804	**0.005**
Postoperative L4 lordosis	−0.223	0.8	0.682–0.94	−2.711	**0.007**
Postoperative PT	0.137	1.147	1.039–1.266	2.725	**0.006**

The bold values are provided in statistical significance.

To further construct a cage subsidence score (CSS) that is convenient for clinical use, as shown in Table 4, based on the OR value of the above regression analysis, we assigned 2 points for the postoperative intervertebral space height, 3 points for postoperative L4 lordosis angle and −4 points for postoperative PT. The total score ranges from −4 to 5.

**Table 4:** Cage subsidence risk scoring table.

Factors	Cutoff value	OR	Scores
Postoperative dis height	>14.68	0.593	2
Postoperative L4 lordosis	>16.91	0.8	3
Postoperative PT	>22.69	1.147	−4

**Figure 4:**
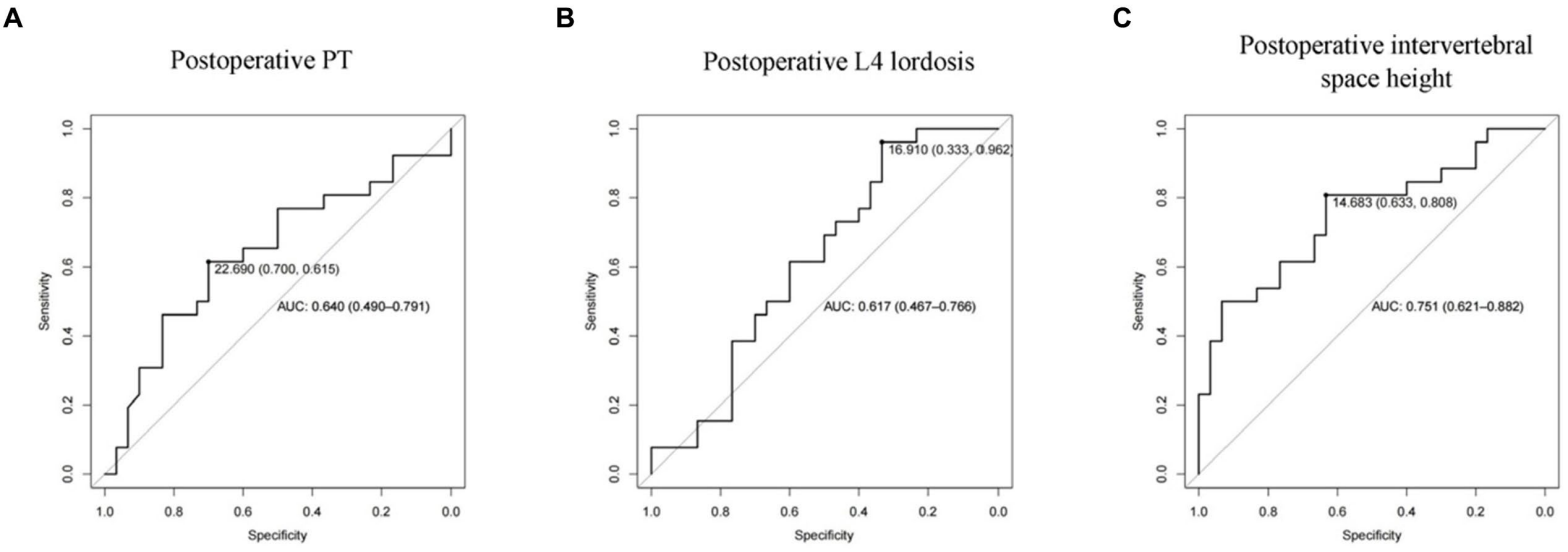
ROC curve of subsidence-related risk factors. (**A**: Postoperative PT; **B**: Postoperative L4 lordosis; **C**: Postoperative dis height).

To determine the reasonable cut-off value for each risk factor, we performed ROC curve analysis on postoperative PT, postoperative L4 lordosis angle, and postoperative intervertebral space height, respectively. The results are shown in Figure 4. The AUC of postoperative PT is 0.640 (0.490–0.791) and postoperative L4 lordosis angle AUC is 0.617 (0.467–0.766), and postoperative intervertebral space height AUC is 0.751 (0.621–0.862). These results indicate that these factors can independently predict the risk of cage subsidence in patients to a certain extent, but their predictive accuracy is not ideal. Finally, the optimal cut-off value for risk factors is determined according to the ROC curve shown in Figure 4 and Table 4, namely, 2 points for postoperative intervertebral space height > 14.68, and 3 points for postoperative L4 lordosis angle >16.91 and postoperative PT > 22.69 was assigned −4 points.

**Figure 5:**
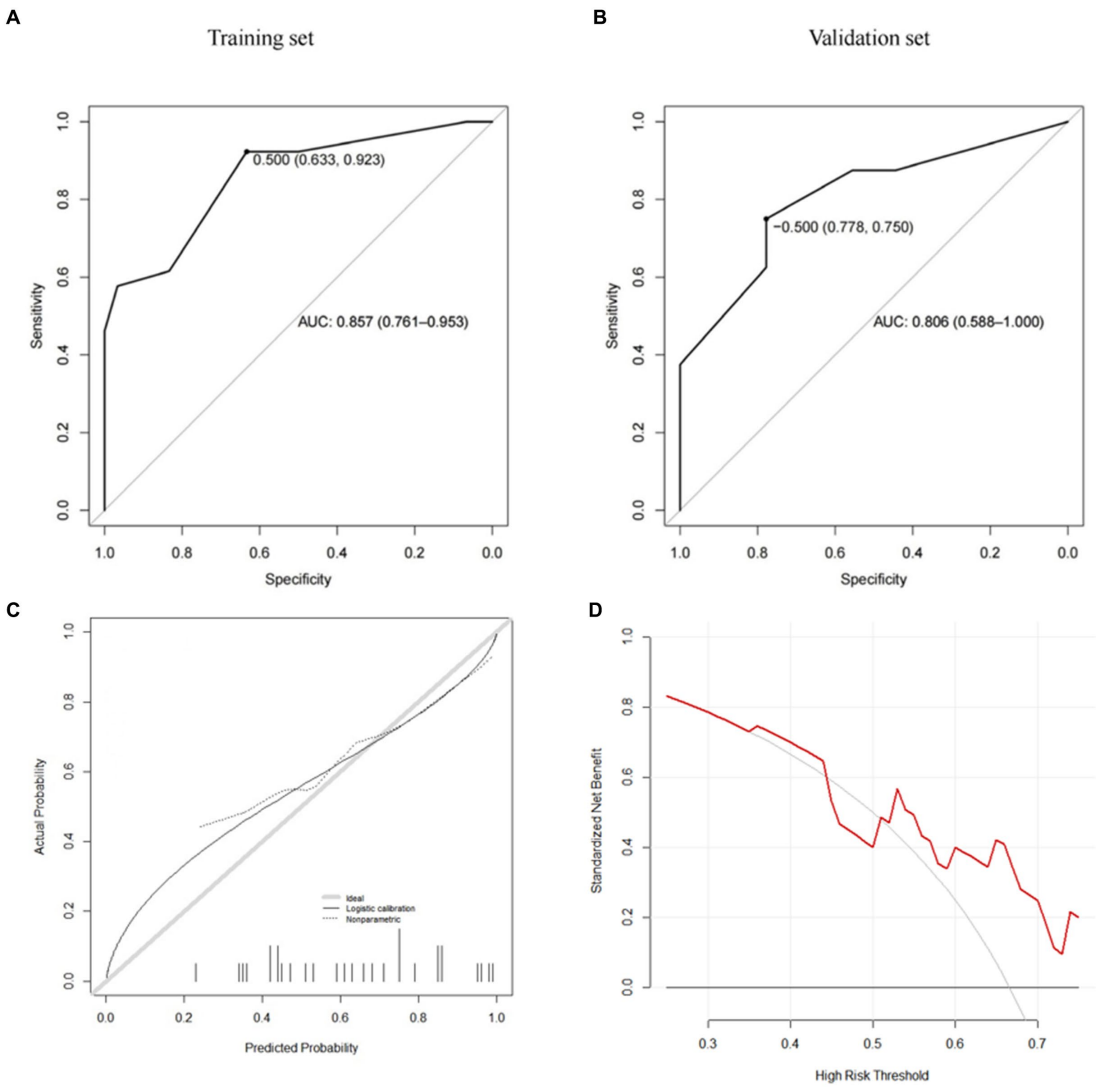
CSS (**A**: training set, **B**: validation set, **C**: calibration curve, **D**: decision curve analysis).

Further predictive performance tests show that the scoring model could more accurately predict the risk of postoperative cage subsidence in patients. As shown in Figure 5 and Table 5, the AUC of the CSS score in the training set is 0.857, the sensitivity is 0.923, the specificity is 0.633, the positive predictive value was 0.686, the negative predictive value was 0.905, and the F1 value is 0.787. In the validation set, its AUC is 0.806, the sensitivity is 0.875, the specificity is 0.556, the positive predictive value is 0.636, the negative predictive value is 0.833, and the F1 value is 0.737. According to Figure 5A, when the score cutoff value is set to 0.5, it has the best prediction performance, so the subsidence risk is considered to be high (overall rate: 88.89%) when the total score is greater than 0.5, and is considered to be low (32.61%) when it is less than 0.5. And the calibration curves of the CSS illustrated consistency between the observed and predicted results (Figure 5C). As shown in Figure 5D, decision curve analysis (DCA) curve indicated that the CSS can serve as a precise tool for subsidence assessment.

**Table 5:** Prediction efficiency of settlement risk score CSS in the training set and verification set.

Evaluation indicators	Training set	Validation set
AUC	0.857	0.806
Sensitivity	0.923	0.875
Specificity	0.633	0.556
Positive predictive value	0.686	0.636
Negative predictive value	0.905	0.833
F1 value	0.787	0.737

### 3.3. Comparison of machine learning model and CSS scoring performance

**Figure 6:**
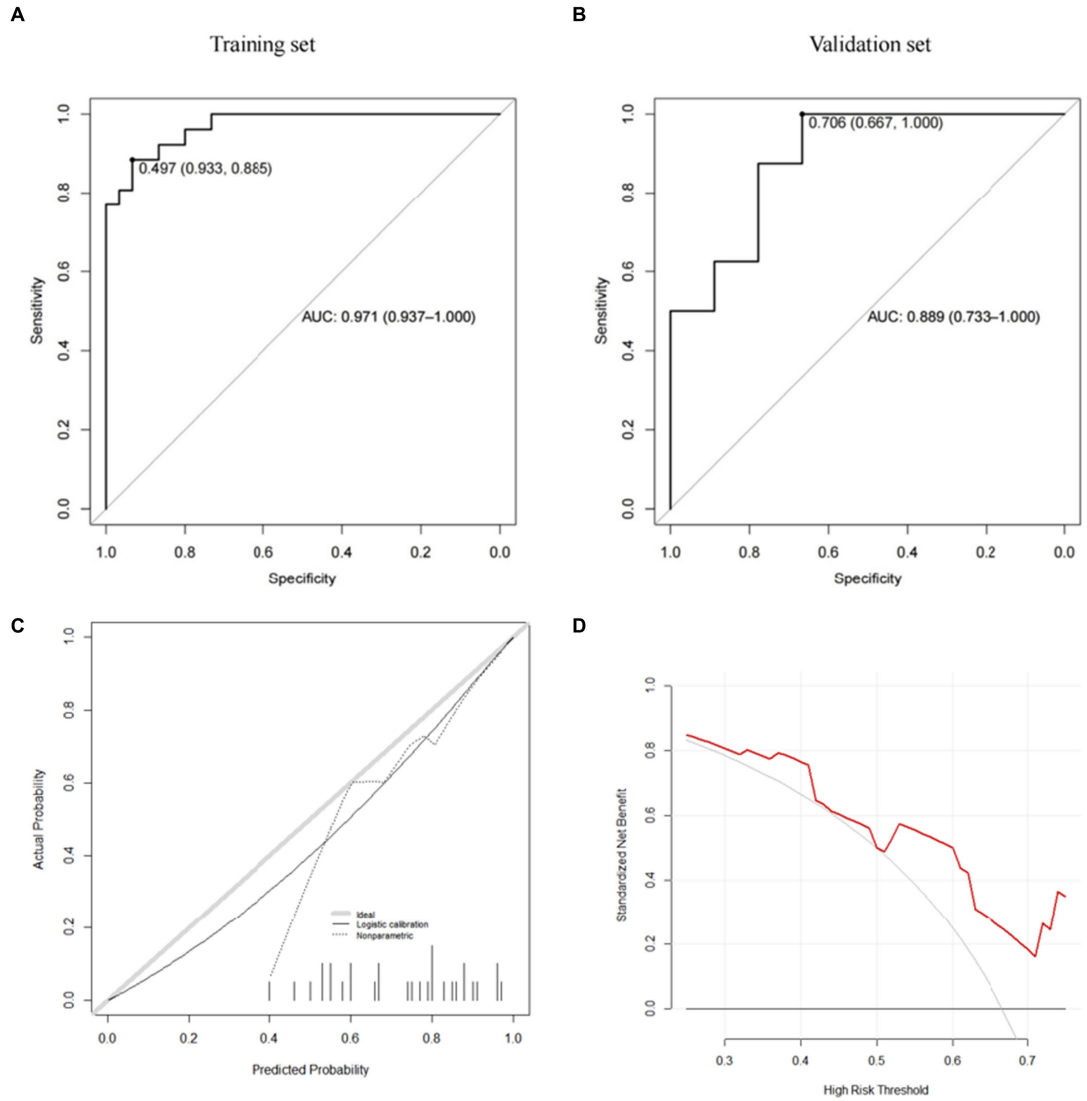
GBM model (**A**: training set, **B**: validation set, **C**: calibration curve, **D**: decision curve analysis).

**Figure 7:**
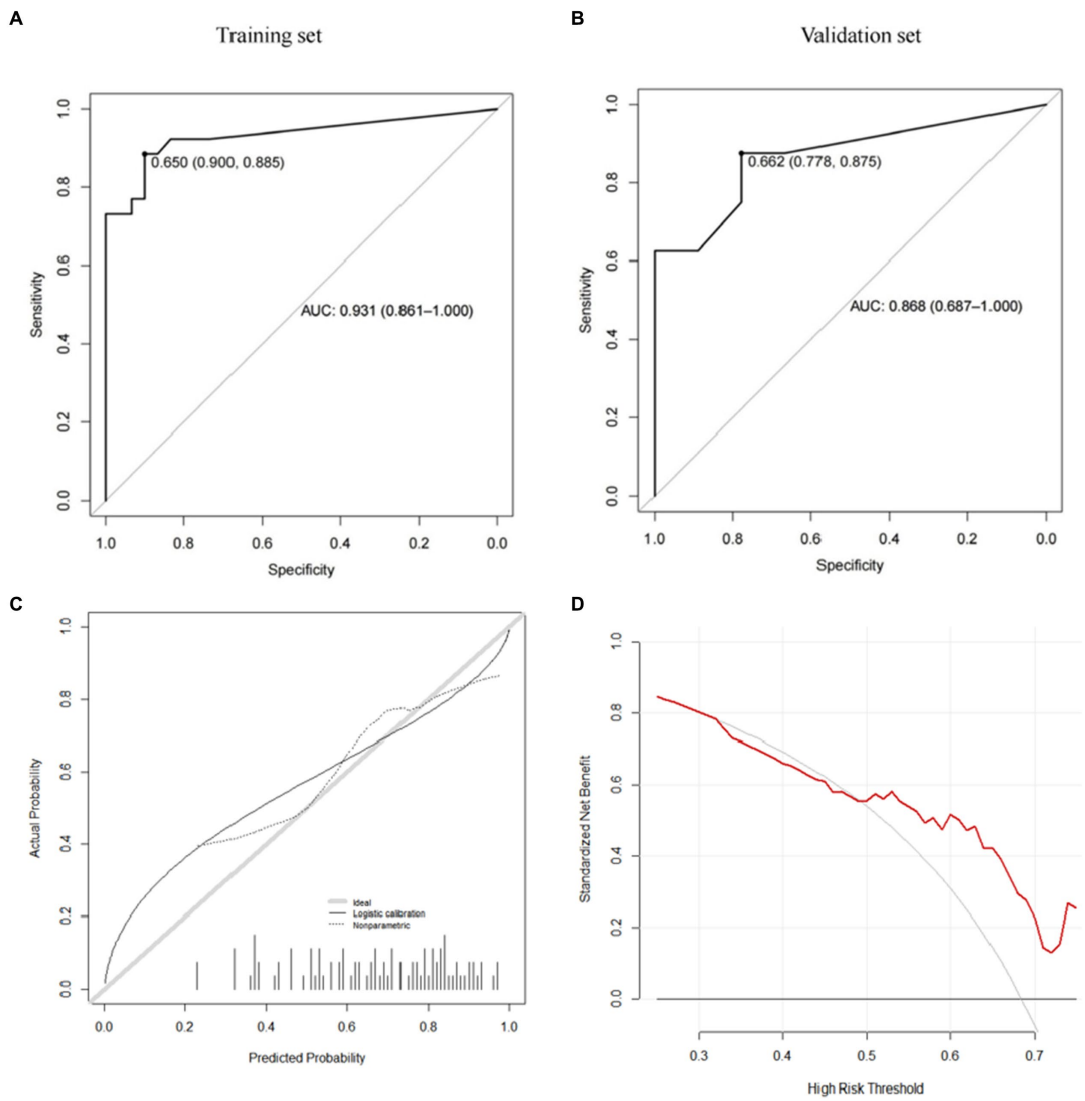
avNNet model (**A**: training set, **B**: validation set, **C**: calibration curve, **D**: decision curve analysis).

Further, we use the caret package to build the risk prediction model of the Gradient Boosting Machine (GBM) and the Model Averaged Neural Network (avNNet). Our results show that both GBM-based and avNNet-based risk assessment models can achieve accurate subsidence risk prediction. As shown in Tables 6, 7 and Figures 6, 7, the AUC of the GBM model in the training set and the validation set are 0.971 (0.937–1.000) and 0.889 (0.733–1.000) respectively, and 0.931 (0.861–1.000), 0.868 (0.687–1.000) in the avNNet model which is all larger than the performance of CSS scores (Table 5, training set: 0.857, validation set: 0.806), where the Delong test indicates that the AUC increment of the GBM model in the training set is statistically significant compared with the CSS score (*p* = 0.004).

**Table 6:** Comparison of parameters of a prediction model in the training set (*n* = 56).

Evaluation indicators	GBM model	avNNet model
AUC	0.971	0.931
Sensitivity	0.885	0.885
Specificity	0.933	0.9
Positive predictive value	0.92	0.885
Negative predictive value	0.903	0.9
F1 value	0.902	0.885

**Table 7:** Comparison of various parameters of the prediction model in the validation set (*n* = 17).

Evaluation indicators	GBM model	avNNet model
AUC	0.889	0.868
Sensitivity	0.75	0.875
Specificity	0.778	0.778
Positive predictive value	0.75	0.778
Negative predictive value	0.778	0.875
F1 value	0.75	0.824

As shown in the analysis of the ROC curve in Figures 6A, 7A, the prediction score of the GBM model >0.497 and c > 0.650 are considered to have a higher risk of subsidence, which belongs to the high-risk subsidence group. At this optimal cutoff value (Tables 5, 6), the sensitivity of the GBM model training set is 0.885, specificity 0.933, positive predictive value 0.920, negative predictive value 0.903, and F1 value 0.902. The sensitivity of the validation set is 0.750, specificity 0.778, positive predictive value 0.750, negative predictive value 0.778, and F1 value 0.750. The training set sensitivity of the avNNet model is 0.885, specificity 0.900, positive predictive value 0.885, negative predictive value 0.900, and F1 value 0.885. The sensitivity of the validation set is 0.875, specificity 0.778, positive predictive value 0.778, negative predictive value 0.875, and F1 value 0.824. The calibration curves of the GBM and the avNNet both illustrated consistency between the observed and predicted results (Figures 6C, 7C). As shown in Figures 6D, 7D, DCAs were performed and the results proved that they both can serve as an effective tool for prediction. And the Delong test indicates that the AUC of the GBM model is no statistically significant compared with the avNNet model (*P* > 0.05).

These results suggest that, under certain circumstances, machine learning-based prediction methods are expected to be more accurate in predicting the risk of subsidence in post-fusion patients than scoring tables based on logistic regression analysis.

## 4. Discussion

Cage subsidence is a common clinical complication after spinal fusion surgery, Tempel et al. believe (52) that cage subsidence can be used as a predictor of postoperative revision, and patients with high-grade cage subsidence had significantly higher rates of postoperative revision compared with patients with low-grade cage subsidence. This suggests that cage subsidence is closely related to the postoperative prognosis. Therefore, it is necessary to identify the risk factors related to the occurrence of cage subsidence in patients after interbody fusion, to conduct a more accurate assessment of the risk of postoperative complications in patients before surgery, and to take effective plans to prevent the occurrence of postoperative cage subsidence.

However, even though multiple previous studies have identified lots of risk factors associated with postoperative cage subsidence, such as postoperative intervertebral space height (53), the number of surgical segments (54), and sagittal spine-pelvic balance (55), suggesting that that the subsidence of the cage is determined by multiple risk factors. However, due to the lack of means for joint assessment of multiple comprehensive factors, previous risk factor studies could not accurately determine the risk of subsidence in patients, which hindered the identification of patients with a high risk of subsidence and reduced the practical clinical application value of relevant research. In this study, machine learning and a logistic regression model are used to predict the postoperative subsidence risk of patients by combining multiple risk factors. It helps to identify patients with a high risk of subsidence, to realize the early detection and prevention of interbody cage subsidence, and reduce the incidence of postoperative complications. Further research and verification will promote “intelligent medicine” and “precision medicine” in spinal surgery at home and abroad.

In this study, univariate analysis showed that preoperative/postoperative vertebral space height, postoperative L4 lordosis angle, postoperative PT and postoperative SS had significant statistical differences. These 5 significant variables were included in stepwise regression analysis, and based on AIC criterion, it was concluded that postoperative PT, postoperative L4 lordosis angle and postoperative vertebral space height were independent predictors, with OR values of 1.147, 0.8, and 0.593, respectively. As is known to all, when the OR value is greater than 1, this variable can be regarded as a contributing factor to the outcome, and the outcome index we set is no subsidence. In other words, the risk of cage subsidence will decrease when PT increases within a certain range. However, when the OR value was in the range of 0 to 1, this variable could be regarded as an obstacle to the occurrence of outcome, that is, larger postoperative L4 lordosis angle and postoperative vertebral space height would increase the probability of subsidence.

Postoperative PT, postoperative L4 lordosis angle and postoperative vertebral space height were analyzed by ROC curve, and the AUC of postoperative vertebral space height was the largest, which was 0.751, indicating that it had the most predictive effect in this study. With regard to the height of the postoperative intervertebral space, Yang et al. (56) pointed out that the excessive extension of the intervertebral space could lead to the subsidence of the fusion cage, Le et al. and Malham et al. recommended the use of a cage with a height of 8 ~ 12 mm in lumbar intervertebral fusion (57, 58). In our study, the cage height used was also 8 ~ 12 mm, and the average height of the postoperative intervertebral space in the subsidence group was (15.92 ± 2.92) mm, higher than the maximum height of the fusion cage used 12 mm, indicating the existence of postoperative overextension of the intervertebral space. Due to the biomechanical imbalance during compression during surgery, the L4 lordosis angle in the subsidence group was larger than that in the non-subsidence group, and the difference was statistically significant, which further indicated that there was overextension or insufficient anterior compression in the vertebral space. We further hypothesized that it was the injury to the endplate during intraoperative cage placement. On the one hand, based on the anatomical basis that the lumbar anterior intervertebral disc height is higher than the posterior intervertebral disc height, it is difficult to implant a cage with a forward angle from the rear. Therefore, more intervertebral space is extended, and the posterior endplate will always bear greater stress during the implantation of the intervertebral fusion device, which is easy to cause posterior endplate injury and increase the risk of the fusion device subsidence. On the other hand, during intraoperative intervertebral compression, the posterior intervertebral space is subjected to more compression force than the anterior intervertebral space, which may lead to insufficient compression force in the overstretched anterior intervertebral space, resulting in reduced contact area and stress concentration between the cage and the endplate, increasing the risk of fusion cage subsidence. Therefore, we suggest that the height of the intervertebral space be measured before surgery to avoid the subsidence caused by blind over-extension during surgery. Intraoperative fusion device implantation should be performed on the decompression side first, and then on the non-decompression side to facilitate implantation. Choose a cage with appropriate specifications. Do not forcibly place the fusion device to avoid damaging the upper and lower bony end plates of the rear space. After implantation of the cage, the decompression side should be pressurized first and the non-decompression side should be pressurized again to ensure that the fusion device is not loose or displaced. However, excessive pressure should not be applied to avoid the concentration of compressive stress in the rear due to insufficient pressure in the front clearance.

With increasing attention to patient-related outcomes, there is increasing evidence that appropriate sagittal alignment during lumbar fusion improves outcomes. In our research results, postoperative PT in the sedimentation group was significantly smaller than that in the non-sedimentation group, and postoperative L4 lordosis in the subsidence group was significantly larger than that in the non- subsidence group. In the normal lumbar spine, where the pelvic morphology is associated with varying degrees of lordosis. Excessive intraoperative pressure can result in a larger lordosis angle, and the larger the lordosis Angle, due to the need for sagittal balance, the body tilts the pelvis backward to reduce the PT to maintain balance. As mentioned above, excessive pressure is often associated with endplate injury, which increases the risk of postoperative subsidence. According to our ROC analysis of postoperative L4 lordosis Angle, the probability of subsidence in this study will increase significantly when the postoperative L4 lordosis Angle is >16.91°. Therefore, we suggest that clinicians should pay more attention to the rod bending angle during surgery, and should not over-correct the lordosis to increase the risk of subsidence.

The main innovation of this study is reflected in the synthesis of previous studies on the risk factors related to cage subsidence and a method for comprehensively evaluating the risk of cage subsidence was established based on machine learning and a logistic regression algorithm.

There are certain limitations in this article: this is a retrospective study and the evidence is not as strong as in prospective cohort studies. Cage subsidence after lumbar interbody fusion is related to many factors. BMI, body function, osteoporosis, etc., due to the long case history and lack of clinical data of patients, the above indexes could not be obtained. Moreover, the sample size of this study was small. We will include those additional indicators in future studies to obtain more reliable results. This study only proves that both machine learning and CSS score can effectively predict the risk of cage settlement after surgery, and preliminarily suggests that the prediction model based on machine learning may have advantages in some indicators. However, it is only a single center study and need an external validation in the future.

## 5. Conclusion

Overall, the results of this paper show that both machine learning and CSS scores can accurately predict the risk of postoperative cage subsidence. Under specific circumstances, the machine learning algorithm is able to achieve a more accurate postoperative risk assessment. These results preliminarily confirm the application value of machine learning in solving practical clinical problems and provide a convenient CSS risk score for the risk assessment of cage subsidence after interbody fusion as well. These results will provide preliminary application experience for the future organic combination of precision medicine, intelligent medicine, and surgical practice.

## Data availability statement

The raw data supporting the conclusions of this article will be made available by the authors, without undue reservation.

## Ethics statement

The studies involving human participants were reviewed and approved by this study was conducted in accordance with the Declaration of Helsinki (as revised in 2013) and was approved by the Institutional Ethics Board of the First Affiliated Hospital of Chongqing Medical University. The patients/participants provided their written informed consent to participate in this study. Written informed consent was obtained from the individual(s) for the publication of any potentially identifiable images or data included in this article.

## Author contributions

TX and BW: writing, original draft preparation, methodology, and formal analysis. TX, BW, WQ, and LY: data curation and editing. TX and YO: supervision and critical review. All authors had read and agreed to the final manuscript.

## Conflict of interest

The authors declare that the research was conducted in the absence of any commercial or financial relationships that could be construed as a potential conflict of interest.

## Publisher’s note

All claims expressed in this article are solely those of the authors and do not necessarily represent those of their affiliated organizations, or those of the publisher, the editors and the reviewers. Any product that may be evaluated in this article, or claim that may be made by its manufacturer, is not guaranteed or endorsed by the publisher.

## Footnotes

^1^ https://cran.r-project.org/
