# Supplementary figures and images for: Development and validation of a risk prediction model for cage subsidence after instrumented posterior lumbar fusion based on machine learning: a retrospective observational cohort study
Source: Front Med (Lausanne). 2023 Jul 21;10:1196384. doi: 10.3389/fmed.2023.1196384 (PMC10401589; doi:10.3389/fmed.2023.1196384)

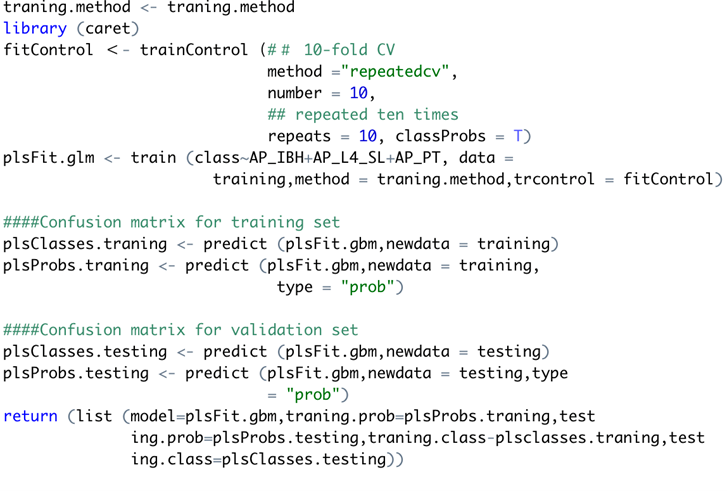

Supplement: Supplementary file 1 [file Image_1.png]
